# Espectrometría de masas en los laboratorios clínicos de proteínas

**DOI:** 10.1515/almed-2024-0071

**Published:** 2024-06-04

**Authors:** Carmen Mugueta, Alvaro González, Sara Deza, Cristina Agulló Roca, Teresa Contreras, Noemí Puig, Nerea Varo

**Affiliations:** Departamento de Bioquímica, 16755 Clínica Universidad de Navarra, Pamplona, España; 37479 Hospital Universitario de Salamanca, Salamanca, Castilla y León, España

**Keywords:** espectrometría de masas, mieloma múltiple, gammapatías monoclonales

Joseph John Thomson fue un ingeniero y matemático inglés descubridor del electrón, que recibió el Premio Nobel de Física en 1906, el mismo año en que Santiago Ramón y Cajal recibía el de Medicina. Thomson ya describió en 1899 un instrumento parecido a un espectrómetro de masas. Fueron sus discípulos, Aston y Dempster, de la Universidad de Chicago, quienes construyeron en la década siguiente los primeros espectrómetros de masas tal y como se conocen en la actualidad. Desde entonces, la tecnología ha avanzado de manera extraordinaria, primero con la introducción de instrumentos de tiempo de vuelo o cuadrupolo. El *electrospray* resolvió después el problema de la ionización de proteínas de gran tamaño y amplió el rango de análisis, previamente restringido a compuestos pequeños. En su conferencia por el Premio Nobel de Química en 2002, Fenn, se refirió a esto como dotar de “alas de electrospray a elefantes moleculares”. Estas mejoras y otras posteriores como el *Matrix Assisted Laser Desportion/Ionization* (MALDI) y la trampa iónica, han convertido a la espectrometría de masas (EM) en una herramienta analítica potente, versátil, precisa y sensible cuyo uso se ha extendido a ámbitos muy diferentes, hasta finalmente llamar también a las puertas del Laboratorio Clínico. Hasta ahora, su uso en rutina en los laboratorios clínicos se ha restringido al análisis de fármacos, hormonas esteroideas y otros metabolitos. Sin embargo, por sus características, el espectro de potenciales aplicaciones de la EM es muy amplio. De hecho, en los últimos años, su uso se ha extendido al análisis de moléculas más grandes como las proteínas, incluyendo la inmunoglobulina monoclonal empleada como biomarcador para el diagnóstico y seguimiento de las gammapatías monoclonales (GM).

El paradigma de GM maligna es el mieloma múltiple (MM), que representa el segundo cáncer hematológico más frecuente. El MM se debe a una proliferación excesiva de células plasmáticas que generalmente producen y secretan una inmunoglobulina monoclonal detectable en el suero y/o la orina y cuya cantidad se considera reflejo de la carga tumoral. En los últimos años, se han introducido fármacos y estrategias terapéuticas (inhibidores de proteasoma, inmunomoduladores, dosis altas de quimioterapia seguidas de trasplante autólogo …) que junto con la inmunoterapia (anticuerpos monoclonales, biespecíficos y células CAR-T) han mejorado sustancialmente el pronóstico del MM. Hoy en día es frecuente que, especialmente con la primera línea de tratamiento, estos pacientes alcancen la mejor respuesta posible en base a los métodos convencionales, que es la “respuesta completa” (RC). Por definición, se alcanza RC cuando la proteína monoclonal (PM) es indetectable mediante electroforesis e inmunofijación en suero y orina y en la médula ósea (MO) se identifican <5 % de células plasmáticas clonales. A pesar de que con los nuevos tratamientos las tasas de RC han aumentado de manera importante, es llamativo que los pacientes siguen recayendo, lo que obviamente se debe a la persistencia de enfermedad que es indetectable empleando los métodos convencionales de evaluación de la respuesta. A esta enfermedad “más allá de la RC” se ha llamado “enfermedad mínima residual” (EMR). De acuerdo con las recomendaciones del *International Myeloma Working Group* (IMWG) la EMR debe estudiarse en pacientes que hayan alcanzado RC.

Dentro de la MO, la EMR puede analizarse a través de la identificación de células plasmáticas tumorales residuales mediante citometría de flujo de alta sensibilidad (*Next Generation Flow*) o de técnicas de secuenciación masiva (*Next Generation Sequencing*) y si es negativa, se debe estudiar la posibilidad de enfermedad fuera de la MO (o extramedular) mediante PET/TC. En 2016 el IMWG publicó los criterios de “EMR negativa” por cada una de estas técnicas con el fin de homogeneizarlas y poder comparar resultados de diferentes tratamientos. Numerosos estudios posteriores han demostrado que alcanzar EMR negativa es uno de los factores con mayor valor pronóstico en MM (tanto al diagnóstico como en recaída) y que se asocia con una supervivencia significativamente más larga, tanto libre de progresión como global.

A pesar del indiscutible valor clínico del análisis de la EMR en MO o mediante PET/TC, estas técnicas tienen también algunas limitaciones. El análisis de la MO mediante NGF o NGS requiere la obtención de una muestra de MO mediante aspirado (o biopsia), un procedimiento invasivo, doloroso y relativamente caro que no puede repetirse con la frecuencia necesaria para llevar a cabo una monitorización adecuada. Además, el análisis se limita a una única muestra, que puede no ser representativa de la celularidad medular y la carga tumoral real (por hemodilución, por la típica infiltración parcheada del MM o porque la afectación sea extramedular) y, por tanto, puede ofrecer en ocasiones resultados falsamente negativos. La PET/TC, tampoco puede repetirse con mucha frecuencia para evitar una irradiación excesiva del paciente, interpretar sus resultados es difícil y no está completamente estandarizado y también son posibles falsos negativos por defectos enzimáticos en las células plasmáticas tumorales que impiden la captación del trazador. Por estas razones, urge investigar la utilidad de muestras alternativas como la sangre periférica, que pueden obtenerse con facilidad y que analizadas con la frecuencia optima mediante métodos de muy alta sensibilidad ofrezcan resultados con valor clínico superior a los convencionales y comparables o complementarios al análisis de la MO.

De esta necesidad clínica surge la aplicación de la EM para la detección de la PM en el suero, técnica que mejora las prestaciones analíticas de los métodos que se emplean hoy en el laboratorio clínico con este fin [1, 2]. La EM puede alcanzar una sensibilidad incluso 1,000 veces superior a la de los métodos electroforéticos y también una especificidad muy alta, por el propio fundamento de la técnica y porque la relación masa/carga de la PM es única y específica de cada paciente. Las utilidades inmediatas de la EM para el diagnóstico y seguimiento de pacientes con MM y otras GM son varias. En primer lugar, es capaz de diferenciar entre la PM tumoral endógena del paciente y los anticuerpos monoclonales terapéuticos, que a día de hoy forman parte del tratamiento de todos los casos con MM. Estos anticuerpos monoclonales terapéuticos, en su mayoría del tipo IgG kappa, pueden interferir con la PM del paciente y dificultar o impedir una correcta evaluación de la respuesta. Aunque se dispone de métodos que permiten eliminar específicamente las interferencias producidas por determinados anticuerpos terapéuticos no es factible disponer de un método diferente para cada potencial anticuerpo monoclonal terapéutico interferente en un laboratorio clínico. La EM, que se fundamenta en la identificación de los compuestos en base a su relación carga/masa específica permite diferenciar inequívocamente entre los anticuerpos monoclonales terapéuticos y la PM endógena del paciente, aportando por tanto una solución universal a este importante reto analítico.

La segunda utilidad de la EM se basa en su mayor sensibilidad para identificar en suero la presencia de una PM comparada con los métodos convencionales [2, 3]. Estudios recientes han demostrado que en aproximadamente un 20 % de los pacientes negativos mediante los métodos estándar (o en RC) aún puede identificarse la presencia de la PM mediante EM y, lo que es más importante, esto se asocia con menor supervivencia libre de progresión. De hecho, el IMWG admite el uso de la EM en lugar de la IF, tanto en práctica habitual como para pacientes en ensayos clínicos [4]. Comparada con la NGF en MO, la EM ha demostrado una sensibilidad discretamente inferior pero un valor clínico comparable que en determinados momentos del tratamiento podría aportar información complementaria al análisis de la EMR en MO. Aunque se necesitan más datos en este sentido, es posible que los resultados de la EM, que por llevarse a cabo en muestras de suero puede repetirse con más frecuencia, ayuden a determinar el momento óptimo para evaluar la EMR, reduciendo así el número de aspirados medulares y optimizando la utilidad clínica de sus resultados. Se ha publicado también que la EM permite identificar la presencia de una PM y monitorizar su evolución tras el tratamiento en pacientes considerados “no secretores” empleando las técnicas convencionales. Esto, en caso de confirmarse e implantarse, facilitaría mucho el seguimiento de estos casos (que de otra manera debe llevarse a cabo con aspirados de MO y/o con PET/TC) y permitiría que estos pacientes pudieran incluirse en ensayos clínicos, de los que habitualmente se excluyen por no tener enfermedad medible.

En tercer lugar, la EM puede aportar información adicional acerca de la PM de los pacientes con MM y otras GM, que no se obtiene mediante los métodos estándar y que tiene valor clínico. Concretamente, puede detectar la presencia de glicosilación de la cadena ligera como modificación post-traduccional, lo que se considera un factor de riesgo de progresión en casos con GMSI y orienta a ciertos diagnósticos como la amiloidosis de cadenas ligeras o la enfermedad por aglutininas frías.

La detección de la PM por EM en pacientes con MM y otras GMs es una opción atractiva dentro de las herramientas del laboratorio por la evidencia que apoya su valor clínico durante todo el curso de la enfermedad. Sin embargo, su implementación no está tampoco exenta de desafíos. La mayor sensibilidad de la EM con respecto a los métodos electroforéticos convencionales puede traducirse en nuevos diagnósticos, cuyas implicaciones clínicas se desconocen, pero que pueden suponer un aumento de la actividad asistencial y de pruebas de seguimiento. La mayor sensibilidad de la EM se traducirá también en unas tasas de RC inferiores a las obtenidas mediante los métodos convencionales de manera que las comparaciones directas entre “ambas RC” deben ser cautelosas. Finalmente, para que la EM salga de los laboratorios altamente especializados o de investigación y se implemente con solidez en la rutina, se deben continuar los esfuerzos comerciales para conseguir, además de una EM automatizada, optimizada para el análisis de un elevado número de muestras y con un software para la interpretación de los resultados, su implementación de una manera coste-efectiva en los diferentes sistemas de salud. Además, la técnica deberá someterse a los mismos criterios de control de calidad que el resto de pruebas y se requerirán extensos procesos de validación. Los especialistas de laboratorio debemos aprovechar nuestra posición transversal privilegiada y conocimiento técnico para conseguir esta optimización en el flujo de atención del paciente que contribuirá a la mejora de su evaluación clínica y pronóstica.
